# Endoscope-Assisted Submandibular Sialadenectomy: A Review of Outcomes, Complications, and Ethical Concerns

**Published:** 2010-05-21

**Authors:** Poramate Pitak-Arnnop, Niels Christian Pausch, Kittipong Dhanuthai, Kraison Sappayatosok, Pichit Ngamwannagul, Ute Bauer, Robert Sader, Alexander D. Rapidis, Christian Hervé, Alexander Hemprich

**Affiliations:** ^a^Department of Oral, Craniomaxillofacial and Facial Plastic Surgery, Faculty of Medicine, University Hospital of Leipzig, Leipzig, Germany; ^b^Laboratory of Medical Ethics and Legal Medicine, Faculty of Medicine, University Paris 5 (René Descartes), Paris, France; ^c^Department of Oral Pathology, Faculty of Dentistry, Chulalongkorn University, Bangkok, Thailand; ^d^Department of Oral Surgery and Oral Medicine, Faculty of Dentistry, Srinakharinwirot University, Bangkok, Thailand; ^e^Department of Oral and Maxillofacial Surgery/Dental Hospital, Faculty of Dentistry, Naresuan University, Phitsanulok, Thailand; ^f^Department of Oral, Craniomaxillofacial and Facial Plastic Surgery, Faculty of Medicine, Medical Center of the Johann Wolfgang Goethe, University Frankfurt am Main, Frankfurt, Germany; ^g^Department of Maxillofacial/Head and Neck Surgery, Greek Anticancer Institute, Saint Savvas Hospital, Athens, Greece.

## Abstract

**Objectives:** To review outcomes and complications of endoscope-assisted submandibular sialadenectomy (EASS) and to analyze this innovative technique with regard to ethical issues. **Methods:** We used a systematic review study design to identify clinical studies on EASS, published in English, French, German, and Thai. The last electronic search was conducted in September 2009. We checked the bibliographies of the identified articles, relevant local journals, and congress abstracts. Publications were further assessed and assigned their respective levels of evidence. We also investigated reporting on human subject protection, conflicts of interest, funding support, and commercial relationships. **Results:** Five case series reporting a total of 28 patients met the inclusion criteria. There was no need of recourse to open surgery. All of the authors claimed satisfactory cosmetic results. Complications were uncommon. However, no controlled trial was available, and outcome measures varied between studies. Human subject protection and funding sources were mentioned in only 2 articles. Commercial relationships and conflicts of interest could not be identified. **Conclusions:** All of the reports favor outcomes of EASS. However, their level of evidence is low, and the superiority of this procedure over the conventional surgery remains unknown. The success of this procedure should not be overemphasized in information for consent and mislead surgeons to begin it without adequate training and elaborate environment. The lack of ethical documentation creates a high degree of suspicion of the studies.

Informed consent becomes important in current medical practice. Clinical decision making without patient participation is considered unethical and, probably, illegal. Inadequate patient consent may lead to discontent and lawsuits. Of several aspects of consent, the risks of the procedure are necessary to be informed preoperatively. In general, surgeons discuss specific and common complications of surgery based on their own experience. However, the consent process is cumbersome when a surgeon begins a new procedure or innovation or is unfamiliar with a novel intervention.^1^

Even though neoplasia constitutes a small proportion of submandibular salivary gland diseases, its consequences are devastating. Surgery is the definitive treatment of a majority of submandibular salivary gland tumors. Conventional open operation satisfies most cases, but it carries various risks, including conspicuous scar, injury to facial, lingual and hypoglossal nerves, infection, fibrosis, and hemorrhage.^2^^-^4

In 1990, endoscopy was initially used in intracorporeal lithotripsy of salivary calculi by Königsberger et al.^5^ Since then, it has been introduced in the fields of craniomaxillofacial and facial plastic surgery. Several advantages are documented, especially decreased tissue damage and surgical complications, improved esthetic results, and fast recovery.^6^^-^8 Based on the first author (P.P.)'s experience, endoscopy is applicable very well to various procedures, including repair of orbital wall fractures,^9^,^10^ excision of the submandibular salivary gland,^11^ and lipomas on the forehead.^12^ Endoscopic removal of dermoid cysts of the neck requires much training and delicate skill (unpublished yet). Nowadays endoscopy is an adjunct in submandibular sialadenectomy in many surgical centers. Nevertheless, outcomes and complications of endoscope-assisted submandibular sialadenectomy (EASS) have not been studied by an evidence-based approach.

The aims of this article were to review reported outcomes and complications of EASS to be part of information for consent. We hypothesized that this novel technique would provide better outcomes and fewer complications than conventional surgery. This evidence-based information will be a supplement to personal data of an individual surgeon. The secondary aim was to analyze this innovative surgery with regard to ethical issues. Ethical considerations on EASS are also discussed.

## METHODS

Using a systematic review study design, we searched the Cochrane library, PubMed/MEDLINE, Embase, and Google Scholar, using the search terms: “salivary gland tumor,” “submandibular gland,” “endoscope,” and “sialadenectomy.” Full-length articles in English, French, German, and Thai published until September 2009 were screened to identify clinical studies on EASS. References of the selected articles, relevant local journals, and congress abstracts were reviewed to identify additional reports for inclusion. We rejected (1) studies with a follow-up period of less than 6 months and (2) studies based on postal questionnaire or case note reviews without postoperative examination.

All included articles were classified on the basis of the grading system adopted by the *Journal of Bone and Joint Surgery*^13^ (Table 1). Evaluation of the articles in French, German, and Thai was assured by the native language–speaking authors. Level categorization and data collection were performed by the primary author (P.P.), and uncertainties were resolved by discussion with all authors. If agreement could not be reached, advice was sought from a third party.

The methodology to identify secondary outcomes of this study was based on our previous research designs.^14^^-^16 One secondary outcome was author's statements on human subject protection (obtaining informed consent and ethics committee's approval) and conflicts of interest in all eligible articles. Reporting of human subject protection was justified, if both processes (obtaining inform consent and ethical approval) were mentioned, or authors explained why one or both were unnecessary. When there was no ethical committee available (as in some developing countries) and a statement of adherence to international research standards (eg, the Declaration of Helsinki) was made, the ethical documentation was considered to be strict.

Another secondary outcome measure was funding sources of the research. Lastly, we investigated the disclosed author-sponsor relationships, which were tabulated as follows: advisory board, consultant/honoraria, educational activities/speakers, employment, grants, family connection, expert testimony, patent/licenses and stock ownership. Authors who had an industry address were categorized as employees.

The recommendations of the Declaration of Helsinki were thoroughly maintained during this study. Because this study did not involve human participants or records, it was exempt from the review by the ethics committee and consent from the authors of the original articles. Data were summarized and evaluated by means of descriptive statistics.

## RESULTS

Five articles were identified and classified in Level IV. The number of cases and length of follow-up were limited in most series. In total, 28 patients underwent EASS without the need of recourse to open means, and complications were uncommon. Zero degree and/or 30°, 4- or 5-mm rigid endoscopes were used. There was no study compared EASS with the conventional operations. Esthetic outcomes were all judged by the innovator authors. Neither an independent outcome assessor nor measure indices of minimally invasive procedures (eg, postoperative pain and surgical blood loss) was mentioned. Details are presented in Table 2.

In 2 articles, the patients were informed that EASS was an experiment, and funding sources were nonprofit organizations. Human subject protection, grant support, and conflicts of interest were not disclosed in the other 3 reports. Commercial relationships could not be identified.

## REVIEW OF LITERATURE

### Conventional and endoscopic submandibular salivary gland surgeries

Submandibular sialadenectomy is indicated in cases of tumors, persistent or refractory sialadenitis, proximally located or intraparenchymal sialolithiasis, and intractable drooling.^11^,^17^,^18^,^21^,^22^ Conventional transcervical surgery provides not only good direct exposure to facilitate safe dissection and a quick operation but possible complications such as visible scar, nerve injury, hematoma, and infection.^2^^-^4,20 To minimize the morbidity, other surgical approaches have been adopted. Submandibular salivary gland resection via an intraoral approach yields promising outcomes. However, it increases the risks of a ranula, salivary fistula, postoperative infection, lingual nerve injury, scar contracture, and subsequent limitation of tongue movement.^22^^-^25 A recent randomized controlled trial revealed the success of submandibular salivary gland excision through a submental approach.^4^

Minimally invasive EASS is another option that shows the most promise. The incision length is reduced from 3 to 6 cm in conventional surgery to 1.5 to 2 cm in EASS. The submandibular gland can be extirpated in one piece through the surgical wound.^11^,^17^,^18^ Although valid measures were not applied, all of the *eureka* authors claimed satisfactory cosmetic results.^11^,^17^^-^20 Good esthetic outcomes may result from minimal stretching or compression of surrounding tissues and full exposure of the gland during the procedure.^18^,^26^

A magnified view of the endoscope enables surgeons to identify and perform meticulous ligatures or diathermy of important structures around the submandibular salivary gland. Injury to neurovascular structures can be minimized.^11^,^17^,^18^ Different devices, including a bayonet-type bipolar cautery device,^18^ an ultrasonically activated scalpel (Harmonic Scalpel®, Ethicon, NJ, U.S.A.),^17^,^20^ or a sheath retractor of the endoscope itself with the aid of endoscopic scissors,^11^ can be used to free the gland. Technical refinement of EASS based on the first author's experience is described in previous publication.^11^ When indicated, computed tomography or magnetic resonance imaging and/or fine-needle aspiration cytology examination of the submandibular salivary gland are made preoperatively.^11^,^16^,^19^ Video records of the procedure are useful for pedagogic purposes.^11^,^26^

The primary tumor of the submandibular salivary gland is almost exclusively pleomorphic adenoma. The slowly expanding tumor attenuates and compresses circumferential tissue of the gland. This normal tissue forms a *pseudocapsule* around the adenoma,^3^,^27^^-^29 creating an avascular plane superficial to the gland during the dissection.^22^,^26^ It should be borne in mind that the pseudocapsule is often incomplete, and pseudopenetration or satellitosis of the tumor is very common.^11^,^27^^-^29 Intraoperative spillage of tumor mucoid causes recurrence of the disease.^20^,^27^,^29^ Therefore, subcapsular dissection that facilitates the ease of the procedure is not desirable in the EASS of pleomorphic adenoma.^11^,^26^,^29^ If the intraoperative capsular rupture occurs, long-term follow-up is recommended because of the higher recurrence rate.^29^,^30^

The important drawback of EASS is prolonged operating time. However, a steep learning curve allows surgeons to overcome the technical difficulties and would shorter the procedure duration.^11^,^17^,^18^,^20^,^22^,^26^ EASS is not suitable for malignant tumors that require neck dissection. When the submandibular gland is inflamed, the EASS schedule should be postponed.^11^,^22^,^26^

## Human subject protection and innovative surgery

Minimally invasive surgery requires adequate training and delicate skill of the surgeons. Formal education with exercises in laboratory, animal or cadaveric models, is necessary before commencing an innovative surgery in humans.^22^ Our recent study and a Cochrane review revealed that articles with positive results received priority in the publication.^16^,^31^ The *publication bias* may make an innovation more glamorous and mislead surgeons to begin EASS without adequate training and elaborate environment. As Clark^32^ reminds us, surgeon's decision to treat can be biased by career self-interest and financial gains. Patients may form an *innovative alliance* by encouraging their surgeons to try any new thing to improve the quality of life or prospects for survival. Meanwhile, surgeons may also be eager to apply that innovation for the same reasons.^32^

An endoscope used in the EASS is akin to that in paranasal sinus surgery.^17^,^18^ However, this does not mean that the endoscopy can be applied to other parts of the human body without risks of damage. Quite clearly, the antral anatomy greatly differs from the anatomical complexity of the submandibular triangle. Even in endoscopic surgery of an organ of the neck, its details diverse from those of other organs of the neck.^26^,^33^ No studies on EASS mentioned ethical approval. The critical questions evolve: “Is a modification of a surgical technique a research?” “Does it require human subject protection?” and “What and how should a surgeon inform the patient?”

Governing bodies such as the US Food and Drug Administration regulate *only* experiments on drugs and medical devices and products, but not reports of a new surgical concept or technique. The boundary between minor modifications and more prominent or extensive alterations of a surgical technique is usually unclear. Case series can therefore be unrecognized as research until they are presented or published.^34^^-^36

Margo^36^ defines *informal research* as a case series that provides clinical parameters and routine follow-up data as study outcomes without a written protocol. It may encompass *no* more than a placebo effect and may be costly, time-consuming, and dangerous to humans. Satisfactory preliminary results can mislead one to add more patients to a study, while risks are invisible until the sample size is large enough.^36^ Innovative surgery is personally specific because it usually comes from talented innovators in a well-equipped environment.^35^^-^38 In this way, a journal would become the media of harm to humans when it distributes danger to other surgeons who lack proper training and equipment.^35^,^36^,^38^ Hence, technical weakness, a degree of safety and efficacy, and factors affecting the learning curve are of great concern when an innovation is introduced.^35^,^36^

Several factors are associated with informal research: ill-defined distinction between a technical variation and an innovation, paternalistic behavior of surgeons, careless research practice, conflicts of interest, and lack of knowledge about research ethics and federal requirements.^35^,^36^,^39^ The US National Commission for the Protection of Human Subjects of Biomedical and Behavioral Research defines the *research's objectives* as “to test a hypothesis, permit conclusions to be drawn, and thereby to develop or contribute to generalize knowledge.”^36^^(p41)^ Therefore, a case series is considered as a subset of human research, necessitating the strict adherence to the ethical requirements and peer review.^40^,^41^

In 1964, the Declaration of Helsinki was adopted after the World Medical Association meeting in Helsinki, Finland and has been amended several times (the 7th revision in 2008). It clearly requires human subject protection. The World Medical Association also invited everyone who designs, conducts, and analyzes research to adopt the Declaration of Helsinki.^42^^-^44 The CONSORT (Consolidated Standards of Reporting Trials) statement does not cover human subject protection. However, the Declaration of Helsinki, the recommendations by the International Committee of Medical Journal Editors, World Association of Medical Editors, Committee on Publication Ethics, International Ethical Guidelines for Biomedical Research involving Human Subjects of the Council for International Organizations of Medical Sciences of the World Health Organization, and in the United States, the Federal Policy for the Protection of Human Subjects (commonly known as the “Common Rule”) do so.^14^,^43^ In France, according to the French laws (*loi n°2002-302* du 4 mars 2002; *loi n° 2004-806* du 9 août 2004), all prospective studies (with the exception of observational studies) and research dealing with human tissue (other than for diagnostic and therapeutic purposes) require ethical approval before beginning the projects. Authors must report ethical processes even if their researches are exempted or waived from the ethical review.^14^,^45^ Surprisingly, an EASS study from France in an oral-maxillofacial surgery journal did not mention human subject protection. This supports our previous study's finding that reporting of human subject protection in oral-maxillofacial surgery researches was less than ideal.^14^

It is prudent to obtain human subject protection if presentation or publication is expected and there is no exemption from local or national regulations.^40^,^41^ Exemption from ethical approval should be decided by the regulatory body or ethical committee, not by researchers themselves, and it should be described in the publication.^14^,^40^,^41^ Failure to maintain human subject protection is ethically unacceptable and regarded as *research misconduct*.^46^

Research involving humans must not be mixed with routine practice and then later reported as a retrospective study. Physicians frequently take on a dual role as investigators: physicians serve the best interests of their patients, but investigators seek truth. These 2 goals are not always compatible.^44^,^45^ Informed consent is not all the same, although the same terminology is used. Clinical consent is information about study results that are standard in the medical community. In research practice, consent describes the hypothesis, methods, and outcomes of a study that may be somewhat unknown. It is therefore challenging for a physician-scientist.^34^^-^36,39,45 To appropriately differentiate between the physician's role as a clinician and an investigator, the American Medical Association advises that consent be obtained by someone other than the *would-be* innovator.^47^

## Patient consent to EASS

As a general rule, patient's permission given under “unfair” or “undue” pressure is not consent.^1^ During the consent process to EASS, the surgeon must be aware of *selective hearing*; patients take all information about potential benefits and filter out all information about potential risks.^32^ The success of the EASS should not be overemphasized, and the surgeon should be risk-averse. Information about the risk/benefit and the standard treatments or alternatives is of paramount importance. However, when a surgeon begins a new procedure, details of the risks and surgical complications are usually uncertain, hampering the consent procedure. Therefore, appropriate data collection from the literature and personal experiences becomes essential.

Because controlled studies are absent, it is impossible to prove the superiority of EASS. The risks of complications may not differ from conventional sialadenectomy, except smaller scars. Hence, EASS seems to be only an option for the selected cases such as in Blacks because of a high incidence of a hypertrophic scar and keloid.^48^ Well-designed studies based on more patients and cost-benefit analyses are still desirable.

EASS candidates should be informed about intraoperative conversion to open exploration in cases of severe adhesion, massive hemorrhage, or frozen section biopsy results indicating malignancy.^17^,^18^,^22^,^26^ If insufflation of the subplatysmal region is performed, additional risks include injury to neurovascular structures, subcutaneous emphysema, air embolization, carotid artery occlusion, tension pneumothorax, hypercapnea, and increased intracranial pressure.^8^,^33^

## Conflicts of interest and clinical surgical research

Concerns about “unduly” influence of conflicts of interest and academic-industry sponsorships on biomedical research are strikingly increasing.^47^,^49^ Conflicts of interest may lead to inappropriate design, conduct, or reporting of research. They threaten scientific integrity by yielding biased study design (eg, inactivating placebo controls) and positive (pro-industry) conclusions, suppressing studies with negative results (denial of access to research results, data holding, publication delay or restrictions), and thus, undermine patient safety and public trust.^47^,^50^,^51^ Financial interests are continuing unabated in a large proportion of medical research and are involved in higher rates of research citations.^47^

Financial interests can be found in individual researchers,^47^,^51^ departmental chairs,^52^ ethical committee members,^53^ editors, and peer-reviewers.^49^ In the United States, the National Institutes of Health and the Food and Drug Administration require that investigators disclose all *significant* financial interests that affect research.^47^ This is in agreement with the International Committee of Medical Journal Editors, World Association of Medical Editors, Committee on Publication Ethics, American Medical Association, and Association of American Medical Colleges. Despite no industry involvement, conflicts of interest must be disclosed.^47^,^49^,^51^^-^53

Commercialism in the EASS studies may occur because of expensive equipment. Our results revealed that the rates of funding and conflicts of interest disclosures were low and failed to evaluate an industrial effect on study outcomes. The possible explanation is that more than 70% of drug trials are funded by industry, while fewer surgical studies have financial support.^50^ Research funded mostly by the government, universities, or professional organizations rarely discloses conflicts of interest.^38^ The lack of ethical documentation may result from the incomplete ethical guidance in the instruction to authors of journals in oral-maxillofacial surgery, plastic surgery, and otolaryngology.^54^ Our recent survey (as yet unpublished) revealed the vast differences in understanding of the research ethics standards among surgical paper authors. Authors may misunderstand research ethics standards, but journals should not ignore this important issue. The credibility of the medical literature requires transparency, not secrecy.^49^

## Study limitations

We are aware of several limitations. First, although we used several searches, our review did not include articles published in other languages than English, French, German, and Thai, or articles in journals not listed by the 4 search engines we used or manuscripts rejected for publication. This might underestimate the number of publications. Second, we did not investigate the actual processes that authors performed human subject protection and used to manage conflicts of interest. The use of an external observer, albeit impractical, would be more accurate to analyze the actual practice. Third, reporting of human subjection protection and conflicts of interest might be absent because authors did not disclose them or because journals did not publish them.

## CONCLUSION

Endoscope-assisted submandibular sialadenectomy (EASS) offers a good surgical view through a minimal cutaneous incision. However, the published studies offer weak evidence. The superiority of EASS over conventional surgery remains unknown because of the absence of controlled trials. The success of the procedure should not be overemphasized in information for consent, and surgeons should be risk-averse. Well-designed studies based on more patients are still required. The lack of ethical documentation indicates a high degree of suspicion of the studies.

## ACKNOWLEDGMENT

This article was completed in partial fulfilment of the requirements for the Double PhD program *Cotutelle de thèse/Doppelpromotion*) to obtain the “Doctorat en sciences” (PhD/DSc) degree at the University Paris 5 (René Descartes) and the “Doktor der Zahnheilkunde” (Dr.med.dent.) degree at the University of Leipzig by the primary author. It is also the collaborative work between these 2 universities.

There was no grant support for this study. The authors indicate full freedom of investigation and no potential conflicts of interest.

## Figures and Tables

**Table 1:** Levels of evidence adopted by the Journal of Bone and Joint Surgery for therapeutic studies^13^

**Level I**	Randomized controlled trial (RCT) with statistically significant difference or no significant difference, but narrow, confidence intervals Systemic review of Level I RCTs
**Level II**	Prospective cohort study, or poor quality RCT (eg, <80% follow-up) Systematic review of Level II studies
**Level III**	Case control study Retrospective cohort studies Systematic review of Level III studies
**Level IV**	Case series (no, or historical, control group)
**Level V**	Expert opinion
**Non-evidence**	Single-case reports Technical notes Animal or laboratory studies Nonsystematic reviews

**Table 2:** Characteristics of the included studies

Authors (year, country)	Level of evidence	No. of patients (gender)	Average age (range, year)	Pathology (No. of patients)	Average operating time (range, min)	Average wound length (range, mm)	Average length of follow-up (range, months)	Average length of hospital stay (range, days)	Complications (No. of patients)
Komatsuzaki et al^17^ (2003, Japan)	IV	4 (4M)	31.3 (24–36)	Intraglandular sialolithiasis (4)	232.5 (175–250)	16.25 (15–20)	?	?	…
Baek & Jeong^18^ (2006, South Korea)	IV	5 (2M, 3F)	26.6 (11–66)	Pleomorphic adenoma (2) Intraglandular sialolithiasis (3)	111.8 (55-180)	22 (15–30)	7.8 (6–11)	?	Severe adhesion to adjacent tissue & lingual nerve injury (1)
Meningaud et al^11^ (2006, France	IV	5 (2M, 3F)	26.6 (16–42)	Pleomorphic adenoma (2) Chronic obstructive sialadenitis (2) Drooling from cerebral palsy (1)	65 (20–120)	? (15–20)	6	0	…
Kessler et al^19^ (2006, Germany)	IV	2 (2F)	43 (23–63)	Chronic obstructive sialadenitis (2)	150 (?)	?	?	?	…
Chen et al^20^ (2006, Taiwan)	IV	12 (7M, 5F)	41.3 (30–59)	Pleomorphic adenoma (3) Sialolithiasis (6) Chronic obstructive sialadenitis (3)	70 (50–125)	23 (20–25)	18 (?)	?	…

M indicates men, F = women, and ? = undetermined.
